# Is the allocation of medical and health resources effective? Characteristic facts from regional heterogeneity in China

**DOI:** 10.1186/s12939-020-01201-8

**Published:** 2020-06-08

**Authors:** Ming Yi, Jiachao Peng, Lian Zhang, Yao Zhang

**Affiliations:** School of Economics and Management, China University of Geosciences, Wuhan, China

**Keywords:** Public medical and health efficiency, Spatial heterogeneity, Three-stage DEA model, Stochastic frontier analysis, I12, I18, H51

## Abstract

**Background:**

Over the last decade, the expenditure on public medical and health has increased greatly in China, however, problems as low efficiency and unfairness still exist. How to accurately describe the effectiveness of existing medical and health resources in combination with regional heterogeneity is of great significance to China’s medical and health reform.

**Methods:**

Based on provincial panel data for the period of 2005 to 2017, combining expected output and unexpected output, this paper constructs a super-efficiency three-stage SBM-DEA model, to measure and analyze the spatial-temporal heterogeneity characteristics and influencing factors of public medical and health efficiency (PMHE).

**Results:**

(1) After the impacts of random error and external environmental factors are removed, the mean value of overall PMHE is 0.9274, failing to reach DEA efficiency, and PMHE shows a fluctuated downward trend. (2) The adjusted PMHE level shows a prominent spatial imbalance at the stage 3. The average efficiency level is ranked by the East > the West > the Central > the Northeast. (3) The increases of GDP per capita and population density are beneficial to the improvement of PMHE, while income level and education level are disadvantageous to PMHE, and last, the urbanization level, an uncertain effect. (4) There is no *σ* convergence of the PMHE in the East, the Central and the West, that is, the internal differences may gradually expand in the future, while the Northeast shows a significant *σ* convergence trending of PMHE. (5) The state’s allocation of medical and health resources has undergone major changes during “The Twelfth Five-Year Plan”.

**Conclusion:**

This study innovatively incorporates undesired outputs of health care into the efficiency evaluation framework by constructing the main efficiency evaluation indicators. The results of the robust evaluation conclude that China’s existing investment in medical and health resources is generally not effective. Therefore, although China’s health care reform has made certain achievement, it is still necessary to expand the investment in health care resources.

## Introduction

In order to achieve the goal of basic medical and health service for all, as well as the improvement of the health of whole nation, a new round of health system reform was initiated by Chinese government in 2009. It was proposed clearly that government would be the main source of public medical and health input, and a government-leading diversified public medical and health input system would be established. The fiscal expenditure on public medical and health had increased from 101.5 billion RMB in 2005 to 1.43 trillion RMB in 2017 with an increase rate of 1313%. The proportion of public medical and health expenditure to total fiscal expenditure had increased from 3.56 to 7.1%. It should be noted that, comparing to developed countries, this input level is still quite low. According to data from *‘Statistical Bulletin of China Health Care Development of 2017*’, the general health care expenditure was about 4.63 trillion RMB in 2016, which accounted for 6.2% of Gross Domestic Product (GDP). According to *World Health Organization* (WHO) statistics, China ranks the 99th among 189 member countries in the ratio of health expenditure to GDP [1]. Moreover, China lacks a sound medical and health service system in that the medical cost goes up very fast, while serious imbalances exist among urban and rural areas. The difficulty of seeing a doctor which the public has been complaining about has not been substantially alleviated. Moreover, the government’s huge investment in health care failed to lighten the direct burden on individuals, and absolute health expense per capita is still rising year by year. The overall personal expenses on health reached 1.49 trillion in 2017, which accounted for 28.8% of total health expenditure.

It can be seen from the previous facts that although the investment in public health care in China has increased significantly, it is still insufficient. The utilization efficiency of medical and health resources is low, that is, insufficient investment and serious waste of resources both exist. Two problems arise from this paradox: (1) Why the public failed to benefit enough from the increase of government public medical and health investment? (2) To increase public health investment, should we prioritize the increase of inputs to catch up with the level of developed countries in the world, or should we focus on creating a balanced and efficient medical and health service system? We believe that the process of medical and health system reform is a complicated social system engineering, although the increase of public medical and health input plays an important role, the improvement of medical and health system operation efficiency, as well as service level and quality are much more vital. Besides, the use of traditional data envelopment analysis in measuring the efficiency value is likely to cause distortion of the result. Therefore, it is necessary to improve the traditional method in order to more accurately measure the real efficiency of the public health care in China, and to find policy possibility to improve the operating efficiency of the public health service system.

## Methods

### Literature review

The public medical and health efficiency (PMHE) is also the efficiency of government fiscal expenditure by nature, namely the economic efficiency. The core of its definition lies in the rationality and validity of resource allocation based on Pareto optimality. In recent years, abundant researches on PMHE have appeared. In these studies, input indicators are normally labor, financial and material inputs, such as government medical and health expenditure, the number of beds in health institutions, medical and health institutions, health personnel, practicing (assistant) doctors, registered nurses and managerial personnel. Different scholars’ studies used different output indicators, but most scholars examined such indicators as life expectancy, infant mortality rate, the number of outpatients, the number of hospital visits, the number of outpatients’ surgeries, the number of inpatients’ surgeries and the number of inpatients’ days [2–9]. For example, Evans et al. choose health expenditure per capita and academic level as input indicators to estimate health system efficiency, and concluded that the health system efficiency varied from completely efficient to completely inefficient [7]. The resources of health systems are critical to improving health condition of people in poor countries, but great gains can be made in most countries by using existing resources more efficiently. Varabyova reviewed the current literatures and synthesized the findings on health system efficiency in OECD countries, and systematically searched five electronic databases in 2014, identified 22 studies that analyzed the efficiency of health care production at the country level [8].

The measurement methods being used are normally parameter method as stochastic frontier analysis (SFA), and non-parameter method as data envelopment analysis (DEA). Grigoli and Kapsoli adopted SFA to study medical and health output efficiency in emerging economic entities, and concluded that the medical and health output efficiency was the lowest in African entities [10]. Berta et al. applied traditional DEA method to measure operation efficiency of hospitals in Italy, and found that the technology efficiency of private hospitals was lower than not-profit public hospitals [11]. Färe and Grosskopf used DEA model to assess medical and health output efficiency of *Organization for Economic Co-operation and Development* (OECD) [12]. Yan made use of DEA-Malmquist index model to estimate the changes of annual efficiency, and intertemporal efficiency of medical and health service in different provinces for the period covering 2009 to 2016 [6]. He also constructed a Tobit regression model to inspect the influence of government medical and health service expenditure on static operation efficiency, dynamic operation efficiency and their elements in different provinces or cities. Taking into consideration the medical demand factors, Zhao measured service efficiency of rural medical institution with a four-stage DEA method in China, and found that big discrepancy existed between efficiencies at both county level and town level [13]. Furthermore, through Free Disposal Hull (FDH), Guptas and Verhoeven used different combinations of single-input and single-output models to estimate the efficiency of health care in 35 countries in Africa, and drew the conclusion that expenditure efficiencies of countries under study were all much lower than European and Asian countries [14]. By means of FDH (Free Disposal Hull) model, Lavado and Cabanda calculated social service expenditure efficiency limited by medical and health, as well as education public resource budget to find that the higher the inequity of resource allocation (measured with Gini coefficient) in the region, the lower the efficiency [15].

As far as the influencing factors are concerned, Pan and Liu suggested that provincial per-capita budget income, provincial population proportion of 15 years old and under, coverage of Burroughs Hospital Information system (BHIS), and urbanization rate were the key factors after assessing real per-capita provincial medical and health efficiency with panel data from 2002 to 2006 in different provinces [16]. Gerring et al. pointed out that economic level, geographic location, education level and epidemic diseases all contribute to public medical and health expenditure efficiency [17]. Li and Wang carried out regression analysis on different factors that could influence PMHE, and found that fiscal decentralization, household registration system, medical and health system reform, urbanization level, economic development level, population density, and education level have significant influence on input-output efficiency in China [18]. Cheng and Liao, Wang et al. believed that fiscal decentralization, population density, together with education level have significant impacts on the efficiency of public health care in China [19, 20]. The difference is that the Chen and Liao believed that both of those two had significant positive impacts, while Wang and Tao believed they had negative effects [19, 20].

In general, traditional DEA method was mainly used by researchers to evaluate medical and health efficiency. Although the research findings are fruitful, two points are missing: Firstly, restricted by model itself, the majority of researches had to select expected output indicators. But due to the fact that some expected output indicators were hard to get, undesirable output indicators were used instead. For instance, the indicator as average life expectancy was hard to get, then undesirable output indicator as human mortality was used instead. Secondly, traditional DEA-CCR model (*Charnes, Cooper, Rhodes*) or DEA-BCC model (*Banker, Charnes, Cooper*) can lead to slack of input factors, resulting in inability to remove random error and influence of external environmental factors on PMHE, and thus lead to efficiency measurement error. Moreover, when there is more than one effective decision-making unit, further comparative studies can’t be carried out.

Aiming at the deficiency of existing researches, this research makes improvement in the following aspects. Firstly, the public medical and health input-output measurement indicators are further improved by including both expected outputs and unexpected outputs at the same time. Secondly, Andersen and Petersen introduced super-efficiency DEA (SE-DEA) model for the first time, which allow efficiency value greater than 1 so that the sequencing of decision-making unit (DMU) could be effectively resolved [21]. Tone proposed Slack-based Measures (SBM) for the first time, in the following year, he combined SE-DEA with SBM, and proposed super-efficiency model to solve the factor slack problem and sequencing problem of effective decision-making unit at the same time [22, 23]. Hereby, this paper adopts super-efficiency three stage SBM-DEA model, and presents a combined super-efficiency model under the assumption of strong disposal situation to identify the quality of effective DMU, to effectively remove random error and disturbance of external factors.

Based on the previous research logic, this paper constructs a super-efficiency three-stage SBM-DEA model with random error and environmental factors removed, and expected output and undesirable output indicators combined, to measure PMHE for 31 provinces in China for the period from 2005 to 2017 so as to discover its spatial-temporal evolution rule and influencing factors. This will provide a feasible method to measure real PMHE in China. In the following sections, public medical and health input-output measurement model will be constructed in Section 3, a comparative study on spatial and temporal evolution rule will be done in Section 4, conclusions and policy suggestions will be shown in Section 5.

### Research design

#### Research method

Traditional DEA model includes CCR model and BCC model [24, 25]. BCC model assumes that returns to scale are changeable, and decomposes the aggregate technology efficiency in CCR model into scale efficiency and pure technology efficiency to solve the effectiveness problem of decision-making unit under changeable returns to scale. The three-stage DEA model was proposed by Fried et al. [26], the biggest advantage of this model lies in the removal of influence of external factors as environmental factors and random factors. In this case, efficiency can be more accurately assessed for more realistic results. This paper combines three-stage DEA model and super-efficiency SBM model to measure PMHE in China. The model estimation is divided into three stages:

At the first stage (stage 1), the traditional DEA model can be used to evaluate the relative efficiency between homogeneous DMUs and to divide the DMUs into two categories, inefficiency and efficiency. The DMUs with the efficiency value less than 1 are inefficient, and the DMUs with the efficiency value of 1 are efficient. However, there are two disadvantages of this method, one is that it is impossible to make further distinction between the efficient DMUs, and the other is that the treatment of unexpected output loses its original economic significance. In the super-efficiency SBM model, not only the unexpected output is properly handled, but also the efficient DMU is accurately distinguished, for example, with the efficiency values of 1.1 or 1.2, an efficiency value of 1.2 means that decision unit efficiency level is higher.

At the stage 1, efficiency values of individual DMU are measured using SBM-DEA model. It is assumed that there are *n* decision-making units which are composed by input *m*, expected output *r*_1_, and undesirable output *r*_2_. With vector representation as *x* ∈ *R*_*m*_, *y*_*d*_ ∈ *R*_*r*1_, *y*_*u*_ ∈ *R*_*r*2_ respectively. *X*, *Y*_*d*_ and *Y*_*u*_ are matrix, where *X* = [*x*_1_, *x*_2_, ⋯, *x*_*n*_] ∈ *R*_*m* × *n*_, *Y*_*d*_ = [*y*_1*d*_, *y*_2*d*_, ⋯, *y*_*nd*_] ∈ *R*_*r*1 × *n*_, and *Y*_*u*_ = [*y*_1*u*_, *y*_2*u*_, ⋯, *y*_*nu*_] ∈ *R*_*r*2 × *n*_. The input matrix is decomposed into the radial part, ${X}^{m_1}\in {R}_{m_1\times n}$, and non-radial part, ${X}^{m_2}\in {R}_{m_2\times n}$, with *m* = *m*_1_ + *m*_2_; the output matrix into the radial part, ${Y}^{s_1}\in {R}_{s_1\times n}$, and non-radial part, ${Y}^{s_2}\in {R}_{s_2\times n}$, with *s* = *s*_1_ + *s*_2_. When discussing SBM, this paper defines decision-making units as effective, so that the following SBM can be established:

1 $$ {\displaystyle \begin{array}{c}\min \rho =\frac{\frac{1}{m}\sum \limits_{i=1}^m\left(\frac{\overline{x}}{x_{\mu }}\right)}{\left(\frac{1}{\left({r}_1+{r}_2\right)}\right)\left(\sum \limits_{s=1}^{r_1}\left(\frac{{\overline{y}}^d}{y_{sk}^d}\right)+\sum \limits_{q=1}^{r_2}\left(\frac{{\overline{y}}^u}{y_{qk}^u}\right)+\right)}\\ {} subject\ to:\left\{\begin{array}{c}\overline{x}\ge \sum \limits_{j=1,\ne k}^n{x}_{ij}{\lambda}_j,i=1,2,\cdots, m;\\ {}{\overline{y}}^d\ge \sum \limits_{j=1,\ne k}^n{y}_{sj}^d{\lambda}_j,s=1,\cdots, {r}_1;\\ {}{\overline{y}}^u\ge \sum \limits_{j=1,\ne k}^n{y}_{qj}^u{\lambda}_j,q=1,\cdots, {r}_2;\\ {}{\lambda}_j\ge 0,j=1,2,\cdots n;\overline{x}\ge {x}_k;\\ {}{\overline{y}}^d\le {y}_k^d,j\ne 0,{\overline{y}}^u\ge {y}_k^u\end{array}\right.\end{array}} $$

At the second stage (stage 2), a similar SFA model is established. It is unavoidable that DMUs will be influenced by environmental factors and random factors. A Similar SFA model can eliminate influence from environmental factors and random factor. Assuming there are *n* DMUs, and there are *m* types of input for each DMU which will be influenced by *p* observable environmental factors, SFA regression was performed for input margin variables of each DMU, and the equation is as follows:

2 $$ {s}_{ik}={f}^i\left({z}_k;{\beta}^i\right)+{v}_{ik}+{\mu}_{ik} $$

In eq. (2), *i* = 1, 2, ⋯, *m*; *k* = 1, 2, ⋯, *n*; *s*_*ik*_ represents input slack variable of the *i*^*th*^ input for the *k*^*th*^ decision-making unit. Among *z*_*k*_ = (*z*_1*k*_, *z*_2*k*_, ⋯, *z*_*pk*_), there are *p* environmental factors, *β*_*i*_ is undetermined coefficient of environmental factor, *f*^*i*^(*z*_*k*_; *β*^*i*^) represents influence of environmental variables on input slack variables with a common representation as *f*^*i*^ = (*z*_*k*_; *β*^*i*^) = *z*_*k*_ × *β*^*i*^; *v*_*ik*_ + *μ*_*ik*_ is the combined error term; *v*_*ik*_ is random disturbance term, ${v}_{ik}\sim {N}^{+}\left(0,{\sigma}_{vi}^2\right)$; *μ*_*ik*_ is administration inefficiency term, ${\mu}_{ik}\sim {N}^{+}\Big({\mu}_i,{\sigma}_{ui}^2$). Assuming that both the above two terms are independent and unrelated, it is defined that $\upgamma ={\sigma}_{ui}^2/\left({\sigma}_{ui}^2+{\sigma}_{vi}^2\right)$, when γ is closer to 1, it means environmental factors plays a dominant role, and when γ is closer to 0, it means random error play a dominant role. In order to adjust the measurement unit to the same environmental factors and random factors, basing on the most effective measurement unit with input volume as the base, the adjustment is shown in eq. (3):

3 $$ {\hat{x}}_{ik}={x}_{ik}-\left[{\mathit{\max}}_k\left({z}_k{\hat{\beta}}^i\right)-{z}_k{\hat{\beta}}^i\right]+\left[{\mathit{\max}}_k\left({\hat{v}}_{ik}\right)-{\hat{v}}_{ik}\right] $$

In eq. (3), two square brackets put all DMUs under the same environment and opportunity, the first of which represents same environmental situation, while the second of which represents same random error situation.

At the third stage (stage 3), the original input data is replaced by the adjusted input volume from stage 2 with same output data. The super-efficiency SBM model is applied again to measure efficiency, after which, a fairer efficiency value for individual DMU excluding influence from external environment and random error is obtained. In addition, we use the optimal solution, and decompose the hybrid efficiency indicator *ρ* into factors as follows:

4 $$ Input\ radial\ inefficiency:{\alpha}_1=1-\rho $$

5 $$ Input\ nonradial\ inefficiency:{\alpha}_2=\frac{1}{m_2}\sum \limits_{i=1}^{m_2}{s}_i^{NR-}/{x}_{i0}^{NR} $$

6 $$ Input\ inefficiency:\alpha =\frac{m_1{\alpha}_1+{m}_2{\alpha}_2}{m} $$

Where ${s}_i^{NR-}$ expresses the radial change, and ${x}_{i0}^{NR}$ is *x* adjusted by *s.*

#### Variable selection

1. Input variables.

Based on the general theory of PMHE measurement, as well as features of public medical and health input, the indicators system can be established from three dimensions, which are labor input, finance input, and material input.

①Labor input (MIN). The labor input variable of medical and health care refers to the number of medical and health personnel. Most scholars classify medical and health personnel into doctors, nurses, and other medical technicians [27–29]. Refering to Xie’s provincial research, we choose health technical personnel number per ten thousand people as human input indicators [30]. This is because, Chinese health personnel quantity covers doctors, nurses and other technical personnel, and this index can reflect the total number of medical and health personnel in each province.

②Finance input (GE). Most scholars include government fiscal health expenditure or health expenditure into health care financial input indicators [31, 32]. With references to the efficiency of health systems, a scattered picture study based on OECD data, government financial expenditure on health was selected as a financial input indicator [33, 34].

③Material input (MHI). Many studies have included the number of hospitals and the number of hospital beds into the investment indicator system [33, 35], but the number of hospitals did not consider social medical service centers, disease prevention and control centers, etc. Therefore, the number of hospital beds is only a component of physical input, the number of medical and health institutions is thus selected as the material input indicator.

(2) Output variables.

The purpose of public medical and health input is to improve maternal and child hygiene level, and disease control level, to prolong average expected lifespan through the enhancement of medical and health service capability. Accordingly, the following output indicators are selected in this paper:

① Medical and health service level (BU).^1^ Beds utilization rate and overall diagnoses and treatment numbers are used as indicators.
② Maternal and child hygiene level. Due to unavailability of perinatal infant death rate data, this paper selects Maternal mortality rate (MMR) and Under-five child mortality (UCM)^2^ as indicators, both of which can also reflect the maternal and child health level.
③ Disease control level (IIR). There are 39 notifiable infectious diseases in China, within which there are 2 Category-A infectious diseases (plague and cholera), 26 Category-B infectious diseases (SARS, Aids, Virus Hepatitis), and 11 Category-C infectious diseases. The incidence rates are available only for Category-A and Category-B infectious disease incidence, therefore, they are used in this paper to measure disease control level.
④ Unexpected indicators. Life expectancy and death rate are the most used indicators to evaluate the health status of residents. Life expectancy is a comprehensive indicator, which cannot reflect the health status and functional status of the living. Mortality index can reflect the health condition of the population at some point, and the changes of death situation and disease spectrum. Because of the comprehensiveness of life expectancy index and the simplicity of existing statistical data, it is difficult to measure this index. Therefore, the population mortality index is selected to indirectly reflect the per capita life expectancy of the residents. Among the above output indicators, total number of patients, total bed occupancy rate, and birth rate are expected outputs. A higher number of patients or the bed utilization rate indicates a higher service level of medical institutions or a higher birth rate. The level of health care is reflected in the level of maternal and child health care in health care institutions. Therefore, the higher the three indicators, the higher the efficiency of health care. The maternal mortality rate, the Category-A and Category-B Statutory Reported Infectious Incidence and the mortality rate of the population are undesirable outputs. The higher the maternal mortality rate, the lower the level of maternal and child health care in regional health institutions. The higher the two indicators of Category-A and Category-B Statutory Reported Infectious Incidence and mortality, the lower the level of disease control and residents’ health status in regional health care institutions. Therefore, these three indicators will reduce the medical and health efficiency of the regions in different degrees, which are selected as unexpected output.

(3) Environment variables.

Environmental variables should meet the requirement of ‘separation assumption’, which means, only the factors that can directly influence PMHE, and the sample data of these factors won’t be subjectively controlled within a short time period can be selected [36]. Based on the research results, the possible influence of the following five factors on PMHE have been reviewed intensively [37–39]: ①Economic development level (PGDP). Real per-capita GDP is used to represent economic development level, and this indicator is expressed by the ratio of real GDP of each province to its population, converted by CPI of a base year. ②Residents income level (RAI). Average annual incomes of different regions are used as indicators. ③Urbanization level (UL). Urbanization rate calculated by urban population to total population is used to express the indicator. ④Population density (POP).^3^ Population density is normally expressed by population size per squared kilometer. ⑤Education level (SNC). This is expressed by average enrolled students at school every 100 thousand people.

#### Data source and processing

The sample data of this paper covers 31 provinces or cities (excluding Hongkong, Macao and Taiwan) in China for the period from 2005 to 2017. Data of indicators and environmental variables are all from *China Statistic Yearbook*, *China Health Statistic Yearbook* and Statistical Yearbooks of provinces. The statistical description results of variables are shown in Table 1. We can see from the table that there is great difference between the maximum value and minimum value of individual variable. GDP per capita shows the greatest difference with a standard deviation of 24,057.73, the maximum value of which is 128.99 thousand RMB per head in Beijing in 2017, and the minimum value of which is 7.84 thousand RMB per head in Yunnan Province in 2005.

**Table 1:** Descriptive Statistical Results of Variables

Type	Name	unit	abbreviation	Mean	Min	Max
Input indicators	Number of health technicians per 10,000 people	person	*MTN*	65.449	25.430	145.224
	Government expenditure	100 million yuan	*GE*	215.228	5.401	1307.560
	Medical and health institution number	unit	*MHI*	24,447.39	1322.0	81,403.0
Output indicators	Beds utilization rate	%	*BU*	82.116	54.700	100.200
	Total visit to health institution numbers	ten thousand	*DTN*	7579.38	255.064	37,146.4
	Maternal mortality rate	Per 100 thousand people	*MMR*	26.854	1.100	290.350
	Under-five child mortality	‰	*UCM*	19.4526	8.4	39.7
	Infectious incidence rate of category A and B	Per 100 thousand people	*IIR*	266.662	102.480	738.190
	Human mortality rate	‰	*HMR*	5.970	4.210	7.400
Environmental variables	GDP per capital	yuan	*PGDP*	38,182.65	5051.96	1.29 × 105
	Resident annual average income	yuan	*RAI*	15,211.77	3562.41	58,988.00
	Urbanization level	%	*UL*	52.033	20.850	89.600
	Population density	per sq. kilo	*POP*	4.037	0.023	29.445
	Average student number in colleges	people	*SNC*	1711.681	554.300	3564.820

Note: Obs = 403, id = 30

The correlation results between input and output variables are listed (Table 2). It can be seen that there is causal relationship between input indicator and output indicator. We know that a perfect linear correlation between indicators won’t influence DEA evaluation result, and a high degree of correlation between indicators can lead to a distorted DEA evaluation result of DMU. Some literatures pointed out that a positive correlation coefficient between input and output variables under 1% significant level will satisfy DEA requirement [40, 41]. Therefore, the correlation of input and output selected in this paper conform to requirements of DEA efficiency.

**Table 2:** Correlation analysis of input and output variables

Var	*MIN*	*GE*	*MHI*	*BU*	*DTN*	*MMR*	*UCM*	*IIR*	*HMR*
*MTN*	1	0.594^***^	0.235^***^	0.293^***^	0.318^***^	−0.518^***^	− 0.201^***^	− 0.227^**^*	− 0.17^***^
*GE*	0.474^***^	1	0.768^***^	0.602^***^	0.780^***^	−0.662^***^	− 0.14^***^	− 0.441^***^	0.261^***^
*MHI*	0.196^***^	0.691^***^	1	0.415^***^	0.612^***^	−0.398^***^	− 0.071	− 0.281^***^	0.397^***^
*BU*	0.331^***^	0.463^***^	0.399^***^	1	0.576^***^	−0.556^***^	− 0.049	− 0.253^***^	0.163^***^
*DTN*	0.285^***^	0.771^***^	0.501^***^	0.434^***^	1	−0.735^***^	−0.342^***^	− 0.460^***^	0.190^***^
*MMR*	− 0.31^***^	−0.37^***^	− 0.31^***^	− 0.47^***^	− 0.39^***^	1	0.428^***^	0.488^***^	− 0.015
*UCM*	−0.23^***^	− 0.01	− 0.027	− 0.0581	− 0.17^***^	0.423^***^	1	0.343***	0.065
*IIR*	−0.13^***^	− 0.33^***^	− 0.29^***^	−0.19^***^	− 0.29^***^	0.232^***^	0.389^***^	1	−0.29^***^
*HMR*	−0.25^***^	0.209^***^	0.382^***^	0.114^**^	−0.026	−0.082^*^	0.064	−0.315^***^	1

Note: Lower – triangular cells report Pearson’s correlation coefficients, upper – triangular cells are Spearman’s rank correlation; ***p < 0.01, **p < 0.05, *p < 0.1

## Empirical study result

### The empirical study result analysis of super-efficiency SBM-DEA model at stage 1

In this paper, super-efficiency SBM-DEA model is used to measure the public medical and health efficiency of 31 provinces and cities in China from 2005 to 2017 by using MaxDEA software. When environmental factors or random factors are not considered, the overall aggregate mean efficiency is 0.869 in the sample period (Table A1), denoting DEA inefficient. According to the dynamic evolution of the annual comprehensive efficiency value, the comprehensive efficiency value reached DEA effective only in 2005 (1.329) and 2008 (1.006). The aggregate values show a downward trend in fluctuation, which means, the growth in public medical and health input failed to bring about improvement in efficiency. There are 10 provinces or cities with aggregated efficiency values bigger than 1, which are, Guangdong, Hainan, Jiangsu, Shandong, Shanghai, Tianjin, Zhejiang, Jiangxi, Ningxia and Xinjiang. Where seven provinces are in the east of China, one province is in the central and two provinces are in the west. Provinces that are in high need of improving PMHE are Inner Mongolia (0.431), Shanxi (0.452), Heilongjiang (0.523), and Jilin (0.523). Taking a regional view, the mean value of PMHE is 1.108 in the east, and is DEA efficient; while the mean values for the central, the west and the northeast are all below 1, and are DEA inefficient. The efficiency level is sorted as follows: The east > the west > the central > the northeast.^4^ As far as efficiency itself is concerned, the public medical and health service level is the highest in the east, while it is the lowest in the northeast.

It should be noted that the previous measurement results didn’t exclude influence from environmental or random factors, and that it can’t truly reflect the actual situation of PMHE. Therefore, further adjustment and measurements need to be done in the following step.

### SFA regression results and analysis at stage 2

At the second stage, SFA method is used to remove influence on PMHE from environmental factors, random error, and of inefficient administration. In the same environment, PMHE is gotten through the adjustment of original input data. This is done through treating the slack variables of labor, finance, and material as explained variables, while income per capita, urbanization level, population density and education levels as explanatory variables. In order to inspect the influence of these 5 environmental factors on the 3 slack variables, Frontier 4.1 are used, SFA regression result is shown (Table 3). The partial result shows significances of different degrees after test. We can conclude from the result that external environmental factors have certain impact on slack variables in different provinces or cities, in this case, it is important to remove environmental and random factors and adjust the input variables.

**Table 3:** Regression results of SFA at stage 2

variables	labor lack		finance input slack		material input slack	
	coefficient	t-test	coefficient	t-test	coefficient	t-test
constant	−13.5054	− 1.424	−33.456	−1.316	22,085.83^***^	1039.76
*PGDP*	−0.0002	−0.7473	− 0.0013^***^	−3.6203	0.0016	0.0228
*RAI*	−0.0002	−0.2927	0.0049^***^	5.1252	0.0313	0.1871
*UL*	0.5889^**^	2.3386	3.3185^***^	3.7801	−196.2938^**^	−2.2373
*POP*	0.6099	1.3578	−8.2436^***^	−3.7733	−415.1307	−1.2562
*SNC*	−0.002	−0.4929	−0.0076	− 0.5699	5.8126^**^	2.6626
sigma^2	922.117^***^	13.9166	11,987.973^***^	5.4237	1541905^***^	1,541,778
gamma	0.0025	0.1035	0.8936^***^	33.694	0.7457^***^	39.3036

Note: ***, **, * represent significance under 1, 5, 10% significance levels respectively

When investigating the impact of environmental variables on input relaxation variables, if the result of the coefficient is positive, it means that an increase in the value of environmental variables will lead to the increase of input relaxation variables, or a decrease of output will lead to the increase of waste and adverse impact on public medical and health efficiency. If the coefficient is negative, it means with the increase of environmental variables, the slack variables will decrease or the output will increase, which is advantageous to PMHE.

1. PGDP.

The regression coefficients of GDP per capita to public medical and health labor input, and finance input slack variables are both negative, and the regression coefficient to finance input lack variable is significant under 1% significance level. This means the increase in GDP per capita can lead to decrease of public medical and health input slack variable, so that waste will be reduced and PMHE is positively affected. This is in accordance with theory and the facts that the higher the economic development level in the region with more financial revenue, the more likely the health expenditure to be higher. Considering the endogeneity of per capita GDP, this paper also uses the DMSP nighttime lighting data of each province or city as the instrumental variable of per capita GDP. The test results show that the economic development level promotes PMHE.

(2) RAI.

The regression coefficients of average resident income to public medical and health finance and material input slack variables are both positive, and the regression coefficient to finance input lack variable is significant under 1% significance level. This demonstrates that the increase of average resident income will bring about the augment of public medical and health finance input slack variable, which means, with the rise of average resident income, the input utilization efficiency will be reduced, which is disadvantageous to PMHE.^5^ One possible reason is that as residents’ incomes increase, residents will continue to adjust their consumption structure, and the demand structure for health care expenditures will also change. When existing public medical and health service system can’t satisfy medical and health service demand, the output efficiency will be negatively influenced.

(3) UL.

The result shows that the regression coefficients of urbanization level to labor and finance input slack variables are both positive, while it is negative to material input slack variable. All of the above coefficients are significant under 1% significance level, which demonstrates that improvement of urbanization level is highly correlated with labor, finance and material inputs slack variables. The promotion of urbanization level can increase input slack variables of labor and finance, but decrease material input slack variable. This leads to the saving of medical and material resources, and the waste of labor and financial resources. Combinely, the final impact of urbanization level on PMHE is uncertain.

(4) POP.

The regression coefficients of population density to public medical and health finance and material input slack variables are negative, and the regression coefficients of population density to finance slack variable is significant under 1% significance level. This denotes that the higher the population density, the fewer the finance input surplus. Possible reason for the saving is that when the population density is higher in the region, the more prominent scaled economy effect of regional government public medical and health expenditure [42], thereby higher output efficiency of local government public medical and health expenditure.

(5) SNC.

The regression coefficient of education level to material input slack variable is positive, and is significant under 1% significance level.^6^ This shows that the improvement of education level can increase public medical and health material input surplus, and generate waste of public medical and health material resource, thus bring about negative influence on PMHE. This result contradicts the research conclusions drawn [43, 44]. We can possibly attribute to the reason that when the education level is higher, people’s requirements for public medical and health service capability and quality are higher. When existing medical service system fails to provide high quality medical and health service, the gap between demand and supply will be broadened, thus the real output level of public medical and health will be brought down.

Obviously, the estimates of economic development indicators (*PGDP*, *RAI* and *UL*) in the above research results seem to be contrary to the reality, especially the finance input slack and material input slack, but is this really the case? We compared the *National Statistical Bulletin* and the *China Health Statistics Yearbook* found that the country’s medical and health resources allocation and financial investment have decreased significantly in recent years. So, where do these reduced resources go? Development is still the top priority in China at this stage, so the improvement of economic development has “Crowding Out” the growth of medical and health resources. This “Crowding Out” effect comes from the implementation of fiscal policies. At this stage, China’s investment in health care is gradually shifting to “efficiency-driven”, not just relying on the advantages of “large amount” of economic resources, but to make use of the self-improvement, self-supplement and repair of the medical system.

#### The empirical study results after input adjustment at stage 3

**Fig. 1:**
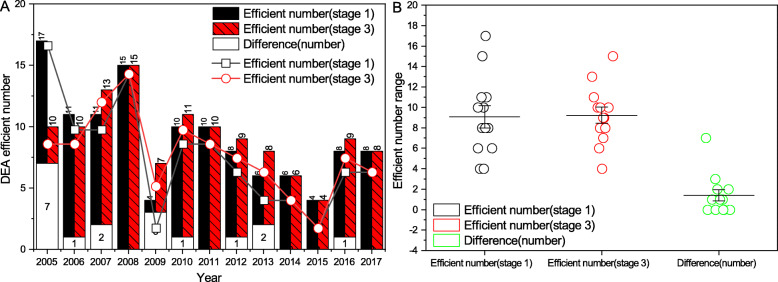
Comparison of numbers of provinces (cities) with efficient DEA at stage 1 and stage 3. **a**, the efficiency value estimates the change trend of DEA effective number. **b**, DEA effective number change interval

At stage 3, adjustments to three input variables are done based on Eq. (3). With the aid of MaxDEA, the adjusted input data obtained from stage 2 and the original output value are put into Super-efficiency three-stage SBM-DEA model again, the efficiency values excluding environmental and random factors are calculated. From the angle of technology efficient frontiers before and after the adjustment, the total number of provinces or cities with comprehensive technology is maintained between 4 and 17, and the efficient figures of VRS are greatly differentiated. After the adjustment, the efficient figures of VRS show a gentler changing trend. The number of the provinces or cities with efficient aggregate technology is 17 before adjustment in 2015, and is 10 afterward. In 2017, the numbers of provinces or cities with input-output efficiency reaching technology efficient frontier are both 8 before and after the adjustment. Detailed figures are shown in Fig. 1a and Fig. 1b.

**Fig. 2:**
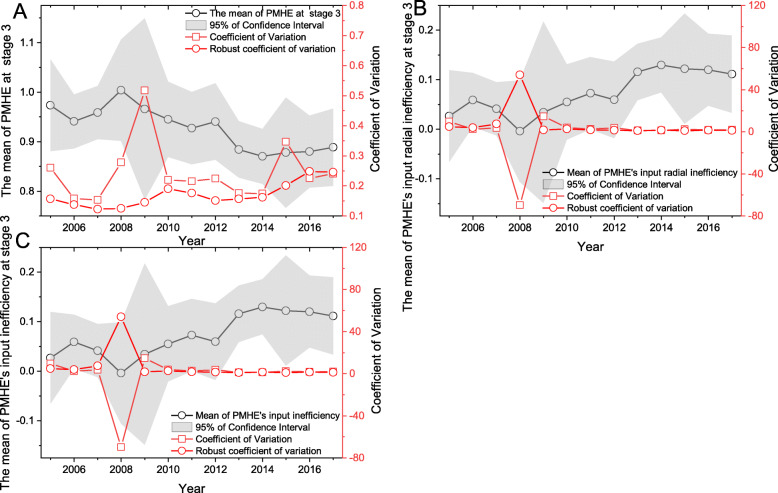
PMHE temporal change trend at stage 3 in 2005–2017. The gray part is 95% of confidence interval. **a**, the mean efficiency of PMHE and its coefficient of variation, robust coefficient of variation at stage 3. **b**, the mean input radial inefficiency of PMHE and its coefficient of variation, robust coefficient of variation at stage 3. **c**, the mean input inefficiency of PMHE and its coefficient of variation, robust coefficient of variation at stage 3

From the measurement results of stage 3 (Table 4 and Fig. 2a), in the sample period, the overall PMHE is 0.927, which is DEA inefficient. Comparing to the optimal region under investigation, there is still room for an increasing of 0.073. Furthermore, from the perspective of the annual report, the comprehensive efficiency value has not been valid for DEA except for 2008 (1.004) (Fig. 2a). The time variation law and spatial distribution characteristics of comprehensive efficiency are further analyzed below.

**Table 4:** The values of PMHE measurement in different regions from2005 to 2017 at stage 3

Region	Provinces	2005	2006	2010	2006–2010	2011	2015	2011–2015	2016	2017	Mean
the East	Beijing	0.819	0.816	0.815	**0.815**	0.881	0.891	**1.017**	1.155	0.984	0.932
	Fujian	0.969	0.965	0.960	**1.018**	0.913	0.908	**0.893**	1.067	1.065	0.973
	Guangdong	1.083	1.221	1.556	**1.216**	1.221	1.275	**1.169**	1.035	1.187	1.172
	Hainan	0.975	0.980	1.078	**1.031**	1.384	2.301	**1.355**	0.976	0.965	1.142
	Hebei	0.968	0.934	0.841	**0.945**	0.924	0.803	**0.887**	0.843	0.844	0.909
	Jiangsu	0.992	1.013	1.135	**1.055**	1.209	1.000	**1.089**	1.077	1.046	1.064
	Shandong	1.142	1.067	1.297	**1.143**	1.011	0.857	**0.922**	1.064	1.020	1.042
	Shanghai	2.128	1.065	1.182	**1.075**	1.303	1.003	**1.231**	1.166	1.448	1.252
	Tianjin	0.823	0.938	1.003	**0.950**	1.166	0.996	**1.032**	1.048	0.842	0.971
	Zhejiang	1.026	1.010	0.972	**1.000**	0.974	0.975	**1.049**	0.977	1.335	1.045
	Mean	1.092	1.001	1.084	**1.025**	1.097	1.101	**1.064**	1.041	1.073	1.050
the Central	Anhui	0.979	0.964	1.019	**0.994**	0.955	0.847	**0.937**	0.910	0.967	0.962
	Henan	0.867	0.854	0.756	**0.818**	0.819	0.825	**0.831**	0.855	0.841	0.832
	Hubei	0.889	0.880	0.868	**0.867**	0.930	0.766	**0.857**	0.791	0.794	0.853
	Hunan	0.967	0.965	0.924	**0.945**	0.924	0.779	**0.851**	0.771	0.752	0.882
	Jiangxi	0.998	1.054	1.210	**1.080**	1.070	0.981	**1.004**	0.913	0.972	1.023
	Shanxi	0.789	0.775	0.655	**0.714**	0.589	0.533	**0.589**	0.544	0.562	0.647
	Mean	0.915	0.915	0.905	**0.903**	0.881	0.789	**0.845**	0.797	0.815	0.867
the West	Gansu	0.813	0.830	0.737	**0.814**	0.717	0.780	**0.759**	0.741	0.758	0.783
	Guangxi	1.013	1.054	0.933	**0.998**	0.830	0.774	**0.827**	0.770	0.836	0.904
	Guizhou	1.059	1.013	0.921	**0.964**	0.872	0.700	**0.773**	0.701	0.717	0.859
	Inner Mongolia	0.738	0.749	0.670	**0.722**	0.652	0.551	**0.611**	0.563	0.568	0.656
	Ningxia	1.247	1.388	1.163	**1.626**	1.041	0.907	**1.075**	1.424	0.975	1.319
	Qinghai	0.985	0.965	0.916	**0.940**	0.878	0.889	**0.908**	0.884	0.858	0.921
	Shaanxi	0.766	0.753	0.724	**0.749**	0.684	0.621	**0.673**	0.610	0.627	0.701
	Sichuan	0.839	0.810	0.922	**0.905**	0.903	0.787	**0.844**	0.799	0.809	0.861
	Tibet	1.111	1.079	1.053	**1.338**	0.876	0.877	**0.903**	0.925	1.142	1.106
	Xinjiang	1.064	0.965	0.974	**1.054**	1.139	0.958	**1.014**	1.026	1.081	1.039
	Yunnan	1.063	0.970	1.032	**1.025**	1.010	0.838	**0.904**	0.861	0.857	0.956
	Chongqing	0.878	0.882	0.933	**0.912**	0.856	0.724	**0.791**	0.719	0.703	0.832
	Mean	0.965	0.955	0.915	**1.004**	0.872	0.784	**0.840**	0.835	0.828	0.911
the Northeast	Heilongjiang	0.724	0.712	0.660	**0.684**	0.672	0.693	**0.698**	0.717	0.676	0.694
	Jilin	0.701	0.725	0.636	**0.691**	0.627	0.675	**0.676**	0.689	0.689	0.686
	Liaoning	0.762	0.769	0.749	**0.760**	0.728	0.697	**0.733**	0.651	0.626	0.731
	Mean	0.729	0.735	0.682	**0.712**	0.675	0.688	**0.702**	0.686	0.663	0.704
Overall		0.973	0.941	0.945	**0.963**	0.927	0.878	**0.900**	0.88	0.889	0.927

Note: 2006–2010 is the mean of PMHE in “The Eleventh Five-Year Plan”, 2011–2015 is the mean of PMHE in “The Twelfth Five-Year Plan”

Due to the existing political system in China, the allocation of medical and health resources has focused on the five-year plan for economic and social construction. Table 4 lists the changes in PMHE during “The Eleventh Five-Year Plan” and “The Twelfth Five-Year Plan” period.^7^ In general, with the gradual improvement of the reform of the medical and health system, “The Twelfth Five-Year Plan” shifted part of the resources in favor of medical and health to economic and social construction, which directly lead to a decline in PMHE during “The Twelfth Five-Year Plan”. This further summarizes some of the conclusions in Table 3. The most representative one is Guangdong Province in the east. Due to the public health crisis caused by the widespread infectiousness of the virus at the beginning of this century during “The Tenth Five-Year Plan”, the central and local governments invested huge financial expenditures in “The Tenth Five-Year Plan” and “The Eleventh Five-Year Plan” to prevent similar public health security incidents, and after “The Eleventh Five-Year Plan”, the central and local governments compressed the original fiscal expenditure on medical and health care. Obviously, this caused PMHE to fall during “The Twelfth Five-Year Plan” period. Although in the early “The thirteenth Five-Year Plan” period, due to the initial success of the construction of the medical and health system, PMHE has rebounded, none have reached the efficiency level of “The Eleventh Five-Year Plan” period.

1. Temporal dynamic evolution rule.

In the sample period, PMHE goes up first and goes down afterward (Fig. 2a). The value increases from 0.973 in the year of 2005 to 1.004 in 2008, and decreases in fluctuation after 2008. Following the Eq. (4) and Eq. (6), we calculate the input inefficiency (Fig. 2b) and input radial inefficiency (Fig. 2c). The input inefficiency had increased from 0.036 in 2006 to 0.131 in 2013 (Fig. 2c), showing a gradual upward trend. The Fig. 2c shows temporal change trend of input radial inefficiency, that is, in the long run, the annual PMHE gap has been expanding.

Figure 2b and Fig. 2c show the consistency of the change trend, but show different object changes: Fig. 2b reflects the input inefficiency trend, indicating the deadweight loss of PMHE in the medical market under the imperfect market, which lead to additional social costs. As PMHE changes, Fig. 2b shows that the corresponding economic and social costs will increase. Figure 2(b) reflects its economic significance, and Fig. 2c reflects the difference between the DMU and the optimal production, which can be reflected in its calculation Eq. (4).

To further illustrate the change trend of PMHE, we calculate the coefficient of variation to analyze the change of PMHE through the *σ* convergence trend, and the results are shown in Fig. 2a.^8^ China’s PMHE has not achieved a state of σ convergence as seen from the Fig. 2a, indicating that in the research sample period, China’s PMHE will be more differentiated. Although the coefficient of variation of PMHE didn’t show significant changes, the increase trend of the robust coefficient of variation showed that PMHE wouldn’t converge at later time.

(2) Spatial distribution heterogeneity characteristics.

In order to analyze the spatial heterogeneity of public medical and health efficiency in different provinces or cities, this paper chooses 1.0, 0.9, 0.8, 0.7, and 0.6 as nodes, classifies efficiency values into 5 intervals, and categorizes the provinces or cities based on their averaged aggregate efficiency values in sample period [40]. There are 10 provinces or cities classified in the first region according to their efficiency values (Table 5), which are Guangdong, Hainan, Jiangsu, Shandong, Shanghai, Zhejiang, Jiangxi, Ningxia, Tibet, and Xinjiang. The PMHE of the above provinces or cities all reach DEA efficiency. Eight provinces or cities have aggregate technology efficiency mean values between 0.9 and 1.0, which are Beijing, Fujian, Hebei, Tianjin, Anhui, Guangxi, Qinghai, and Yunnan. The provinces of Shanxi, Inner Mongolia, Heilongjiang, Jilin have aggregate technology efficiency mean values between 0.6 and 0.7. We can conclude that real PMHE in different provinces or cities are highly differentiated and unbalanced.

**Table 5:** Mean PMHE of provinces or cities from 2005 to 2017

Mean PMHE	DMU			
	the East	the Central	the West	the Northeast
PMHE≥1.0	Guangdong, Hainan, Jiangsu, Shandong, Shanghai, Zhejiang	Jiangxi	Ningxia, Tibet, Xinjiang	
0.9 ≤ PMHE<1.0	Beijing, Fujian, Hebei, Tianjin	Anhui	Guangxi, Qinghai, Yunnan	
0.8 ≤ PMHE<0.9		Henan, Hubei, Hunan	Guizhou, Sichuan, Chongqing	
0.7 ≤ PMHE<0.8			Gansu, Shaanxi	Liaoning
0.6 ≤ PMHE<0.7		Shanxi	Inner Mongolia	Heilongjiang, Jilin

**Fig. 3:**
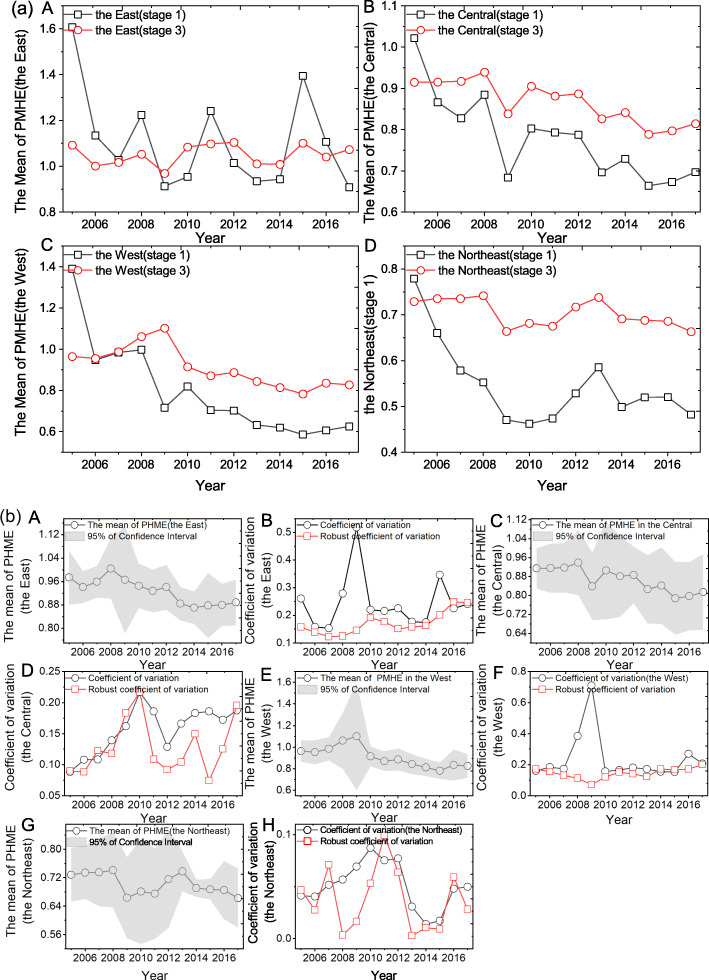
The efficiency means of PMHE in four regions from 2005 to 2017. **a**, Comparison of efficiency means of PMHE for four regions from 2005 to 2017 between stage 1 and stage 3. **a A-D,** the comparison of the efficiency values of the stage 1 and stage 3 in the east, the central, the west and the northeastern. **b**, the mean of PMHE with 95% confidence interval and displayed the coefficient of variation in the four regions. **b A**, the mean efficiency value of PMHE and its 95% confidence interval at stage 3 in the East. **b B**, the trend of the coefficient of variation and the robust coefficient of variation at the third stage in the East. **b C**, the mean efficiency value of PMHE and its 95% confidence interval at stage 3 in the Central. **b D**, the trend of the coefficient of variation and the robust coefficient of variation at the third stage in the Central. **b E**, the mean efficiency value of PMHE and its 95% confidence interval at stage 3 in the West. **b F**, the trend of the coefficient of variation and the robust coefficient of variation at the third stage in the West. **b G**, the mean efficiency value of PMHE and its 95% confidence interval at stage 3 in the Northeast. **b H**, the trend of the coefficient of variation and the robust coefficient of variation at the third stage in the Northeast

As seen from the four major regional levels of the east, the central, the west and the northeast region (Fig. 3(a), we can sequence the aggregate efficiency mean values from high to low as following: the east region (1.050, Fig. 3(a)A), the west region (0.911, Fig. 3(a)B),the central region (0.867, Fig. 3(a)C) and the northeast region (0.704, Fig. 3(a)D). A value of 1.050 shows DEA efficient in the east. The mean values of aggregate efficiency could be improved by 0.089, 0.133 and 0.296 in the west, the central and the northeast, respectively. It shows that the use of public medical and health resources is relatively extensive in these three regions, and the effective development and utilization are insufficient, which means, there may exist problems of “waste of medical and health resources”, and insufficient investment in medical and health resources.

The composition is the time-series of the comparison of the stage 1 and stage 3 of PMHE in the four regions. In these diagrams, the impacts of external environment and random factors are excluded, and the changes in the efficiency of public health in the four major regional sectors all show different trends. In general, the time-varying trend of efficiency in the third phase was more gradual than that in the stage 1. Except the east, the PMHEs of the central, the west and the northeast at stage 3 are higher than that of the stage 1, and the average change in the efficiency of the stage 1 and stage 3 are poor in the northeastern region. The values are bigger than those in the east, central and western regions. (According to Table A1 and Table 4, the mean differences of efficiency before and after adjustment in the eastern, central, western and northeastern regions are − 0.058, 0.088, 0.117, 0.157, respectively).

Referring the Fig. 2, we make a *σ* convergence analysis of PMHE in four major regions, and the results are shown in Fig. 3(b). As can be seen from Fig. 3(b), the CV value of the PMHE change showed a volatility upward trend in the east and the central regions from 2007 to 2017, which indicates that the PMHE did not show a *σ* convergence trend, and that, it has an expanding trend in east and the central area. Although the coefficient of variation of the PMHE fluctuated in the west from 2007 to 2010, it did not show large fluctuations during the sample period. Which means, the widening or narrowing of the internal gap of the PMHE in the western region needs further verification.^9^ During the sample period, the change of PMHE shows a clear *σ* convergence trend in the northeast. Although the changes in PMHE’s internal differences in the above regions are different, the coefficient of variation showed a clear upward trend before 2010. Since then, the change trends in the four major regions have been different, and eventually showed different trends. This shows that PMHE has an obvious time inflection point, which is the year of 2010. This also further demonstrates the changes in the allocation of medical and health resources by the central and local governments during “The Twelfth Five-Year Plan”.

### Robustness test

This paper uses a three-stage method to evaluate China’s PMHE, analyzes the spatial and temporal differentiation characteristics of PMHE and its related influencing factors. Compared with other conventional methods, this paper discusses the robustness of the results from two aspects:

#### Bootstrap test

In order to verify the robustness of the efficiency measurement results, based on the Bootstrap method, SPSS22.0 software was used to measure the confidence interval of efficiency, and the average value of the overall PMHE in China and different regions before and after the input adjustment. In order to improve the reliability of efficiency measurement, frequency of Bootstrap is set to 2000 (Table 6). When influence from environmental factors and random factors are excluded, the aggregate efficiency mean value of China has been greatly improved, an increase from 0.869 to 0.927. Possible reasons could be that the efficiency of all four regions are greatly influenced by environmental factors. When these factors are removed, changes arise: There is a subtle change of aggregate efficiency mean value with a decrease from 1.108 to 1.050 at 95% confident interval in the east; there is certain improvement of aggregate efficiency mean values in the central and the west, an increase from 0.779 to 0.867 in the central and an increase of 0.795 to 0.911 in the west; there is a huge increase from 0.547 to 0.704 in the northeast.

**Table 6:** Aggregate technology efficiency means values and confidence intervals before and after adjustment

Region	Before Adjustment		After Adjustment	
	Mean	Confidence Interval (95%)	Mean	Confidence Interval (95%)
the East	1.108	[0.829,1.386]	1.050	[0.958,1.143]
the Central	0.779	[0.703,0.855]	0.867	[0.827,0.906]
the West	0.795	[0.672,1.063]	0.911	[0.891,1.097]
the Northeast	0.547	[0.489,0.606]	0.704	[0.681,0.727]
Overall	0.869	[0.689,1.048]	0.927	[0.851,1.004]

#### Mean value change of calculation results of different DEA models

**Fig. 4:**
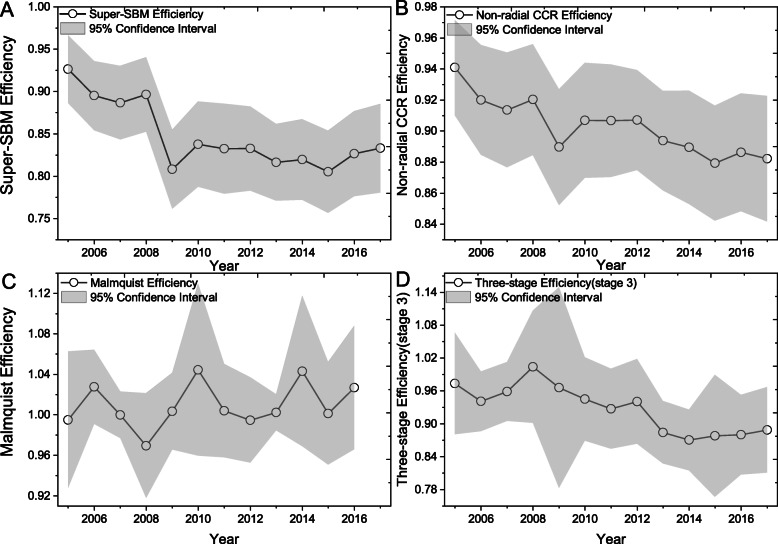
Annual Mean value change of calculation results of different DEA models by Super-SBM, Non-radial CCR model and bootstrap bias, Malmquist model, and this paper used model. **a**, the efficiency was estimated by the super-SBM model and its 95% confidence interval. **b**, the efficiency was estimated by the non-radial CCR model and its 95% confidence interval. **c**. the efficiency was estimated by the decomposition of Malmquist index method and its 95% confidence interval. **d**, the efficiency value calculated by this paper and its 95% confidence interval

**Fig. 5:**
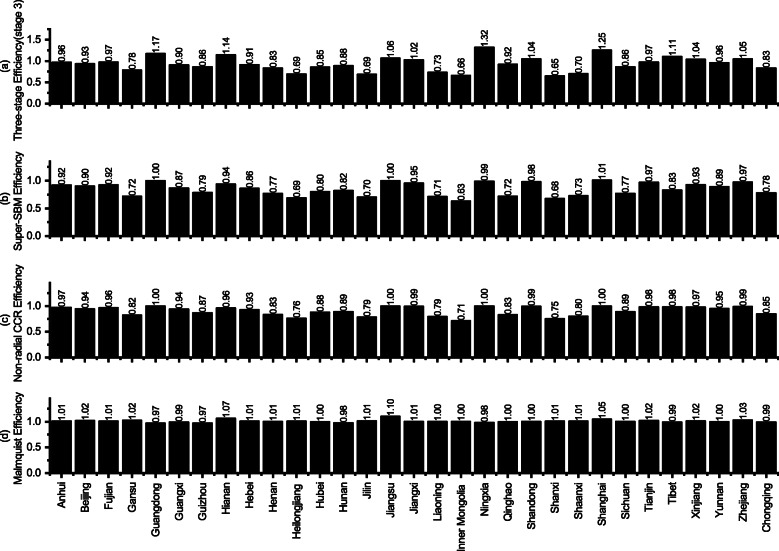
Regional Mean value change of calculation results of different DEA models by Super-SBM, Non-radial CCR model, Malmquist model, and this paper used model. SBM model by Tone and Tsutsui, Non-radial CCR model by Chung et al., the bootstrap bias by Simar and Wilson, Malmquist model (DDF) by Cheng and Zervopoulos [46–49]

In the bootstrap test discussion, this paper compares the confidence intervals of the stage 1 and stage 3, and concludes that the results of stage 3 are more accurate. In addition, this paper selects a representative DEA method to eliminate PMHE and then use the above method to calculate the bias, and then compare the results of the new results with the original calculation results to compare the robustness of the calculation results of various methods. We use three different DEA methods to calculate the PMHE and compare it with the results calculated using the model in this paper. The results are shown in Fig. 4, Fig. 5 and Table A2. Where, the Malmquist productivity index evaluates the total factor productivity change of a DMU between the two time periods. The efficiency change reflects the degree to which a DMU improves or worsens its efficiency, while technological change reflects the change of the efficiency frontiers between two periods. So, we list the efficiency change without technological change and Malmquist productivity index in Fig. 4c.

In Fig. 4, the Super SBM model (Fig. 4a), the CCR model (Fig. 4b), the Malmquist model (Fig. 4c), and the result calculated were used to compare the mean values (Fig. 4d) and calculate the final bias (Table A2). The average annual variation trend of Fig. 4a and Fig. 4b are similar with the result calculated in this paper (Fig. 4d), and the possible reason is that the economic and social cost of Fig. 2b is increased. The results calculated by the Malmquist model generally show an increasing trend, and in Fig. 5(d), the PMHE of all DMUs is valid for DEA efficient. This is obviously different from the factual reflection, and the Malmquist model has the largest bias in Table A2. Therefore, we believe that the results of PMHE are relatively stable. This is mainly because the method used in this paper uses the SFA model estimation of parameter regression in the adjustment process, so the result is more accurate than the non-parametric DEA model [40].

## Conclusions and policy implications

### Conclusions

This paper applies a three stage super-efficiency SBM-DEA model to measure and analyze temporal variation rule, spatial distribution variation of PMHE of 31 provinces or cities from 2005 to 2017, the influencing factors of efficiency and their effects. The findings are listed below:

1. Both the measurement results from stage 1 and stage 3 show that the overall PMHE is descending in fluctuation in China, which means, the increase of public medical and health input failed to bring about PMHE improvement. The real efficiency measured in stage 3 shows that PMHE values are all below 1, except for the year of 2008, which means, there are space for the efficiency to be improved.
2. There is obvious spatial variation of PMHE among different provinces and regions. The real efficiency results from stage 3 show that there are only 10 provinces or cities reach DEA efficient, ample room for efficiency improvement. On the other hand, there exist big differences of PMHE among the four regions. The east has the highest efficiency, followed by the west, the central, the northeast, and the east is the only region with efficiency value above 1. In addition, there is no *σ* convergence in the PMHE in the east, the central and the west, that is, the internal differences may gradually expand in the future, while the northeast shows a significant *σ* convergence trend.
3. When we look at the external factors, it is certain that environmental factors and random factors have greatly influenced PMHE. Improvement of GDP per capita and increase of population both contribute to the improvement of the efficiency. Average resident income and education level are negatively correlated with the efficiency. A higher urbanization level will increase utilization of material input, but decrease utilization of manpower and finance resources.
4. After deducting the influence of external environmental and random factors, the efficiency means values change greatly for all regions in China. After this adjustment, PMHE is improved as a whole, as well as the efficiency of the Central, the West, and the Northeast. We can conclude that environmental and random error factors will affect the real PMHE. Therefore, it is reasonable and necessary to measure PMHE using super-efficiency three stage SBM-DEA model.

### Policy implications

The advantage of this study is that the above empirical evidence can help decision makers to formulate and formulate effective policies to improve PMHE in China. Effective policy tools obtained from the estimated results of PMHE influencing factors can be targeted to areas where the benefits may be greatest.

The policy suggestions include: (1) there is still room for improvement of PMHE in China, no matter at regional, provincial or overall levels. Therefore, while increasing public medical and health investment, there is a greater need to improve medical and health service capabilities and service levels, reduce the amount of slack in input elements, and improve the efficiency and quality of the medical and health service system. Specifically: firstly, reduce the medical and health workforce to accelerate the improvement of economic development level and education level; secondly, in order to reduce the slack amount of medical and health financial elements, it is necessary to accelerate the increase of population density, average education level and economic development level. The low efficiency of PMHE caused by insufficient level of levelization; Finally, in order to reduce the amount of slack in physical factors, it is necessary to increase the level of urbanization and population density, and expand the audience of medical and health resources. (2) considering the significant differences in public health efficiency between different provinces, cities and different regions, differentiated policies should be in place to achieve comprehensive and balanced regional health care development. For example, favorable policies should be given to central, western and northeastern regions to increase support to these areas, and improve the quality of medical and health services in different regions by establishing a sound quality control system for health care, to narrow the PMHE difference between regions. (3) maintaining stable economic growth is an important prerequisite and basis for ensuring the continuous increase of public health care investment. At the same time, it is necessary to further increase the proportion of medical expenditure in total fiscal expenditure. Finally, considering the comprehensive role of urbanization, China should continue promoting the process of urbanization and promote the spatial distribution of population and the spatial distribution of medical and health resources.

The research limitations of this article: First, the relevant indicators adopted in this article are mainly based on the existing literature and input-output framework. Some indicators are subject to discussion. For example, there are many alternative indicators for environmental factors. Under the specific conditions of the SFA model, it is necessary to further test the relevant conclusions through alternative indicators. Second, due to space limitations, there is still the possibility to continue the study of convergence of PMHE, especially the existence of spatial heterogeneity in China. The spatial convergence of PMHE is studied by means of spatial statistical analysis which can be further used to study current status of China’s medical and health resources allocation.

## Data Availability

The datasets used during the current study were available from the corresponding author on reasonable request.

## Appendix A

**Table 7:** The PMHE results from2005 to 2017 at stage 1

Region	Provinces	2005	2006	2007	2008	2009	2010	2011	2012	2013	2014	2015	2016	2017	mean
East	Beijing	0.690	0.637	0.563	0.601	0.542	0.604	0.838	1.000	1.000	1.123	0.850	1.853	1.034	0.872
	Fujian	1.146	1.006	1.144	1.420	0.815	0.955	0.847	0.864	0.656	0.823	0.824	0.980	0.981	0.959
	Guangdong	1.156	1.711	1.087	1.699	0.955	1.000	1.319	1.231	1.174	1.027	1.000	1.000	1.000	1.181
	Hainan	1.154	1.185	1.076	1.660	0.952	1.110	3.065	0.786	0.764	0.826	5.783	0.678	0.560	1.508
	Hebei	0.873	0.848	0.895	1.200	0.757	0.684	0.878	0.841	0.789	0.847	0.711	0.716	0.763	0.831
	Jiangsu	0.993	1.052	1.052	1.181	1.043	1.214	1.000	1.000	1.000	1.000	1.002	1.000	1.000	1.041
	Shandong	1.379	1.337	1.265	1.566	1.179	1.000	1.000	0.892	0.810	0.881	0.794	1.000	1.000	1.085
	Shanghai	6.492	1.208	1.266	1.048	0.967	1.000	1.000	1.000	1.056	1.000	1.036	1.000	1.000	1.467
	Tianjin	1.170	1.370	0.891	0.803	1.038	1.039	1.524	1.306	0.969	0.904	0.973	1.870	0.745	1.123
	Zhejiang	1.007	0.989	1.035	1.063	0.882	0.930	0.936	1.224	1.126	1.000	0.976	0.968	1.000	1.010
	mean	1.606	1.134	1.028	1.224	0.913	0.954	1.241	1.014	0.934	0.943	1.395	1.107	0.908	1.108
Central	Anhui	1.160	0.949	0.984	1.089	0.838	1.009	0.920	0.944	0.830	0.971	0.709	0.803	0.877	0.930
	Henan	0.887	0.773	0.752	0.803	0.582	0.596	0.708	0.745	0.682	0.697	0.698	0.752	0.712	0.722
	Hubei	0.953	0.751	0.755	0.704	0.615	0.746	0.852	0.825	0.701	0.690	0.634	0.632	0.626	0.730
	Hunan	1.439	0.957	0.889	0.852	0.718	0.797	0.784	0.724	0.665	0.647	0.618	0.609	0.598	0.792
	Jiangxi	1.086	1.201	1.032	1.351	0.941	1.232	1.120	0.997	0.917	0.974	0.953	0.855	0.974	1.049
	Shanxi	0.607	0.568	0.554	0.507	0.408	0.435	0.375	0.491	0.383	0.396	0.371	0.387	0.394	0.452
	mean	1.022	0.867	0.828	0.884	0.684	0.802	0.793	0.788	0.696	0.729	0.664	0.673	0.697	0.779
West	Gansu	0.786	0.742	0.646	0.730	0.633	0.545	0.530	0.536	0.624	0.587	0.597	0.548	0.545	0.619
	Guangxi	1.042	1.088	0.975	1.076	0.835	0.825	0.665	0.666	0.736	0.682	0.616	0.642	0.674	0.809
	Guizhou	1.142	1.057	0.978	0.978	0.754	0.794	0.738	0.664	0.556	0.507	0.476	0.462	0.448	0.735
	Inner Mongolia	0.612	0.607	0.569	0.545	0.388	0.408	0.420	0.405	0.368	0.338	0.316	0.321	0.312	0.431
	Ningxia	5.917	0.976	0.995	2.194	1.000	1.852	1.076	1.000	1.000	1.013	0.829	1.000	0.969	1.525
	Qinghai	0.971	0.947	0.931	0.950	0.618	0.629	0.554	0.568	0.523	0.520	0.509	0.501	0.453	0.667
	Shaanxi	0.751	0.667	0.607	0.578	0.481	0.524	0.513	0.568	0.503	0.475	0.456	0.436	0.442	0.539
	Sichuan	0.867	0.809	0.852	0.935	0.833	0.804	0.771	0.746	0.701	0.659	0.641	0.659	0.659	0.764
	Tibet	1.129	1.802	1.000	1.000	0.705	1.030	0.516	0.564	0.557	0.667	0.496	0.601	1.000	0.851
	Xinjiang	1.356	0.740	2.267	1.054	0.709	0.699	1.000	1.218	0.762	0.781	0.903	1.000	1.000	1.038
	Yunnan	1.069	0.967	1.085	1.115	0.977	0.973	1.007	0.844	0.694	0.677	0.628	0.625	0.606	0.867
	Chongqing	1.038	0.956	0.908	0.812	0.671	0.747	0.667	0.647	0.566	0.527	0.565	0.487	0.403	0.692
	mean	1.390	0.947	0.984	0.997	0.717	0.819	0.705	0.702	0.633	0.619	0.586	0.607	0.626	0.795
Northeast	Heilongjiang	0.783	0.589	0.527	0.528	0.435	0.459	0.473	0.496	0.528	0.503	0.492	0.518	0.472	0.523
	Jilin	0.747	0.680	0.599	0.501	0.384	0.372	0.389	0.462	0.613	0.459	0.519	0.545	0.534	0.523
	Liaoning	0.807	0.712	0.608	0.629	0.593	0.557	0.560	0.628	0.615	0.535	0.550	0.498	0.441	0.595
	mean	0.779	0.660	0.578	0.553	0.470	0.462	0.474	0.529	0.586	0.499	0.520	0.520	0.482	0.547
Overall China		1.329	0.964	0.929	1.006	0.750	0.825	0.872	0.803	0.738	0.733	0.856	0.772	0.717	0.869

## Appendix B

**Table 8:** Calculating the PMHE mean value of Bootstrap bias results by different methods

provinces	Super SBM model	Nonradial CCR model	Stage 1	Malmquist model
Anhui	0.051	0.017	0.012	0.322
Beijing	0.062	0.022	0.017	0.345
Fujian	0.073	0.042	0.030	0.320
Gansu	0.043	0.006	0.005	0.108
Guangdong	0.110	0.028	0.022	0.332
Guangxi	0.054	0.018	0.015	0.142
Guizhou	0.056	0.029	0.019	0.223
Hainan	0.081	0.037	0.026	0.414
Hebei	0.054	0.016	0.012	0.155
Henan	0.047	0.012	0.009	0.163
Heilongjiang	0.037	0.006	0.005	0.166
Hubei	0.047	0.009	0.007	0.151
Hunan	0.046	0.013	0.008	0.071
Jilin	0.043	0.008	0.007	0.216
Jiangsu	0.113	0.030	0.025	0.212
Jiangxi	0.062	0.023	0.018	0.311
Liaoning	0.042	0.008	0.006	0.184
Inner Mongolia	0.040	0.003	0.003	0.099
Ningxia	0.092	0.024	0.018	0.251
Qinghai	0.049	0.013	0.011	0.248
Shandong	0.098	0.033	0.025	0.330
Shanxi	0.043	0.003	0.003	0.055
Shaanxi	0.041	0.006	0.005	0.142
Shanghai	0.100	0.024	0.019	0.240
Sichuan	0.039	0.010	0.007	0.112
Tianjin	0.087	0.029	0.019	0.177
Tibet	0.081	0.045	0.036	0.323
Xinjiang	0.065	0.016	0.012	0.183
Yunnan	0.051	0.015	0.011	0.233
Zhejiang	0.073	0.020	0.016	0.284
Chongqing	0.044	0.011	0.008	0.164

## Abbreviations

BHIS: Burroughs Hospital Information system
BU: Beds utilization
CV: Convergence of variation
DEA-BCC: Banker, Charnes, Cooper model
DEA-CCR: Charnes, Cooper, Rhodes model
DEA: Data envelopment analysis
DMSP: Defense Meteorological Satellite Program
DMU: Decision Making Units
DTN: Overall diagnoses and treatment numbers
FDH: Free Disposal Hull
GDP: Gross Domestic Product
GE: Government expenditure
HMR: Human mortality rate
IIR: Infectious incidence rate of category A and category B
MHI: Medical and health institution number
MMR: Maternal mortality rate
MTN: Medical technician number per ten thousand people
OECD: Organization for Economic Co-operation and Development
PGDP: GDP per capital
PMHE: Public medical and health efficiency
POP: Population density
RAI: Resident annual average income
SBM: Slack-based Measures
SE-DEA: Super-efficiency DEA
SFA: Stochastic frontier analysis
SNC: Average student number in colleges
UCM: Under-five child mortality
UL: Urbanization level
VRS: Variable returns to scale
WHO: World Health Organization

## Footnotes

1 This refers to the bed utilization rate (%), and the number of hospital beds refers to the number of beds in medical institutions in “③ Material Input (MHI)”, that is, these beds can be supplied by Medical institutions do their best. The bed utilization rate (%) reflects the ratio of daily bed use to actual bed use, that is, the ratio of the total bed days occupied to the total bed days opened.

2 The infant mortality rate in the monitored areas of *China ‘s Health Statistics Yearbook* includes New born infant mortality rate (‰), Infant mortality rate (‰), Under-five child mortality (‰) and Maternal mortality rate (1/100,000). Considering that the Under-five child mortality (‰) and Maternal mortality rate (1/100,000) are more capable of measuring the output capacity of the region’s medical and health resources about maternal and child hygiene level, it is used as one of the maternal and child hygiene level indicators. Thanks to the review experts for their help in this part.

3 From two independent perspectives of UL and POP, their impacts on PMHE are proposed: is there an alternative relationship between UL and POP? That is, in the case of low UL, can the increase in POP significantly increase PMHE to make up for the problem of low efficiency caused by insufficient urbanization; on the other hand, in the case of insufficient POP, the improvement of UL has made up for the inefficiency of PMHE caused by insufficient POP.

4 The National Bureau of Statistics of China stipulated that since 2005, the specific divisions of the East, the Central, the West and the Northeast involved in the statistics are: the 10 eastern provinces (cities) include Beijing, Tianjin, Hebei, Shanghai, Jiangsu, Zhejiang, Fujian, Shandong, Guangdong and Hainan; 6 provinces in central China include Shanxi, Anhui, Jiangxi, Henan, Hubei and Hunan; 12 provinces (regions and cities) in western China include Inner Mongolia, Guangxi, Chongqing, Sichuan, Guizhou, Yunnan, Tibet, Shaanxi, Gansu, Qinghai, and Ningxia And Xinjiang; the three northeast provinces include Liaoning, Jilin and Heilongjiang. For details, see the website: http://www.stats.gov.cn/tjzs/cjwtjd/201308/t20130829_74318.html.

5 The increase of residents’ income does not make use of capital investment in health care. This is mainly because the current state reform of the public healthcare system relies only on financial burden and allocation of medical resources. The development of China’s medical and health resources mainly relies on large public hospitals, with high barriers to entry, and it is difficult for residents’ income to cross the threshold. In addition, this article mainly discusses public health, which leads to an increase in residents’ income and may lead to investment in private medical resources. Such investment will snatch public medical resources. This high threshold and the obstruction of the political system make even the increase in residents’ income does not effectively promote PMHE, which will be the main topic of our discussion in the next stage.

6 We also tried to use other proxy variables, such as average years of schooling, etc., and the regression results did not differ significantly. Therefore, we mainly discussed the influence and possible causes of this variable.

7 “The Tenth Five-Year Plan” is mainly the tenth five-year plan outline formulated by the Chinese government for economic and social development. It is a grand blueprint for China’s economic and social development from 2001 to 2005. Correspondingly, “The Eleventh Five-Year Plan” is for the period 2006 to 2010, “The Twelfth Five-Year Plan” is for 2011 to 2015, while “The thirteenth Five-Year Plan” is for 2016 to 2020.

8 Convergence of variation (CV) refers to the variation of PMHE between different regions or provinces, which gradually decreases over time. As in refence [45], we use the following equation to analyze the variation trend of the coefficient of variation (CV) of the PMHE in each region.

9 We can further study the internal differences of different regions according to the spatial analysis method, due to space limitations, we will not discuss it further here.
